# The Impact of Chronic Kidney Disease on Oral Health: A Narrative Review

**DOI:** 10.3390/jcm15134940

**Published:** 2026-06-25

**Authors:** Petra Magdalena Kes, Anđela Krndelj, Stella Jurić, Ena Hadžović, Nikolina Bašić Jukić, Vlaho Brailo

**Affiliations:** 1 University of Zagreb School of Dental Medicine, 10000 Zagreb, Croatia; pkes@sfzg.unizg.hr (P.M.K.); akrndelj@sfzg.unizg.hr (A.K.); sjuric@sfzg.unizg.hr (S.J.); ehadzovic@sfzg.unizg.hr (E.H.); 2 Department of Nephrology, Arterial Hypertension, Dialysis and Transplantation, Clinical Hospital Centre, 10000 Zagreb, Croatia; 3 Department of Oral Medicine, University of Zagreb School of Dental Medicine, University Clinical Hospital Centre, 10000 Zagreb, Croatia; brailo@sfzg.unizg.hr

**Keywords:** chronic kidney disease, kidney transplantation, xerostomia, gingival hyperplasia, oral cancer

## Abstract

**Background/Objectives**: Chronic kidney disease (CKD) is associated with numerous oral manifestations that may negatively affect quality of life, nutrition, and overall health. This narrative review aimed to summarize current evidence regarding oral manifestations of CKD and kidney transplantation, examine their proposed underlying mechanisms, and discuss implications for dental management. **Methods**: A structured literature search of PubMed/MEDLINE was conducted for English-language publications from January 2000 to March 2026. Original studies, systematic reviews, meta-analyses, clinical guidelines, and relevant narrative reviews were included. Additional references were identified through manual screening of bibliographies. **Results**: Oral manifestations associated with CKD include xerostomia, periodontal disease, oral infections, anemia-related mucosal pallor, developmental enamel defects, and medication-related gingival overgrowth. Kidney transplant recipients are additionally at risk of opportunistic infections and oral malignancies related to long-term immunosuppressive therapy. While oral diseases, particularly periodontal disease and oral infections, may contribute to systemic inflammation, much of the available evidence remains observational. Similarly, many recommendations for dental management are based on expert consensus and clinical experience rather than high-quality interventional studies. **Conclusions**: Oral complications are common throughout the CKD continuum and warrant regular assessment and preventive care. Multidisciplinary collaboration is essential, while further prospective studies are needed to strengthen the evidence base for clinical management.

## 1. Introduction

The kidneys play a vital role in maintaining overall homeostasis by regulating fluid and electrolyte balance, excreting metabolic waste products, controlling blood pressure, activating vitamin D, and producing erythropoietin [1,2]. Chronic kidney disease (CKD) is characterized by a progressive decline in kidney function and represents a major global health challenge, affecting more than 10% of the world’s population. The prevalence of CKD continues to increase due to population ageing and the growing burden of diabetes mellitus and hypertension, which are the leading causes of the disease [2,3]. As CKD progresses, patients frequently develop systemic complications, including cardiovascular disease, anemia, mineral and bone disorders, immune dysfunction, and metabolic disturbances [1,2]. Many of these systemic alterations manifest in the oral cavity. Patients with CKD may develop a wide range of oral manifestations, including xerostomia, periodontal disease, oral infections, mucosal pallor associated with anemia, developmental dental abnormalities, and medication-related gingival overgrowth [4,5]. Furthermore, individuals undergoing kidney transplantation require lifelong immunosuppressive therapy, which may increase their susceptibility to opportunistic infections and oral malignancies. These oral conditions can negatively affect nutrition, communication, quality of life, and overall health status [6].

Growing evidence suggests a bidirectional relationship between oral health and CKD. While CKD-related metabolic and immunological alterations contribute to oral disease development, chronic oral inflammatory conditions, particularly periodontitis, may increase systemic inflammatory burden and potentially contribute to adverse renal and cardiovascular outcomes. In addition, poor oral health has been associated with increased systemic inflammation, reflected by elevated levels of inflammatory biomarkers such as C-reactive protein and interleukin-6, as well as higher hospitalization rates and reduced quality of life in patients with CKD [6,7]. Despite increasing recognition of this association, oral health remains underrepresented in the routine management of CKD patients. The evidence regarding the prevalence, pathogenesis, clinical significance, and management of oral manifestations is dispersed across different medical and dental disciplines. Consequently, clinicians may face challenges in identifying, preventing, and managing oral complications in this medically complex patient population.

Although numerous studies have investigated individual oral manifestations of CKD, the available evidence remains fragmented across nephrology, oral medicine, periodontology, pediatric dentistry, and transplant medicine. Existing reviews have often focused on specific conditions such as periodontitis, xerostomia, or developmental enamel defects, while comparatively few have provided a comprehensive clinical overview spanning the entire CKD continuum, from predialysis disease and dialysis to kidney transplantation [4,5,6,7]. Furthermore, the strength of evidence supporting proposed associations and management recommendations varies considerably, and many clinical practices are based on observational studies, expert consensus, or institutional protocols rather than high-quality interventional research. Consequently, there remains a need for an updated synthesis that critically summarizes current knowledge, highlights areas of uncertainty, and provides a clinically relevant framework for the management of oral health in patients with CKD and kidney transplantation.

This review was guided by the following research questions:

- What are the most common oral manifestations observed in patients with chronic kidney disease and kidney transplantation?
- What mechanisms have been proposed to explain their development?
- What is the current level of evidence supporting their clinical significance and management?

To address these questions, this narrative review synthesizes current evidence regarding oral manifestations associated with CKD and kidney transplantation, examines the proposed biological mechanisms underlying these conditions, evaluates the strength of available evidence, and discusses their implications for clinical practice and multidisciplinary patient care.

## 2. Materials and Methods

This narrative review was conducted through a structured literature search of the PubMed/MEDLINE database performed in March 2026. The search strategy included the following keywords and their combinations: “chronic kidney disease”, “CKD”, “end-stage renal disease”, “ESRD”, “haemodialysis”, “haemodialysis”, “dialysis”, “kidney transplant”, “renal transplant”, “oral lesions”, “oral mucosa”, “oral disease”, “periodontitis”, “gingivitis”, “gingival hyperplasia”, “gingival enlargement”, “xerostomia”, “dry mouth”, “hyposalivation”, “stomatitis”, “candidiasis”, “Candida”, “oral infection”, “mucosal pallor”, “enamel hypoplasia”, “developmental defects of enamel”, “dental caries”, “lip cancer”, “oral cancer” and “oral malignancies”. The literature search strategy is outlined in Supplementary Table S1.

The search was limited to articles published in English language between January 2000 and March 2026. This search period was selected to focus on evidence most relevant to current clinical practice rather than to identify all historical publications on the topic. Original research articles, systematic reviews, meta-analyses, clinical guidelines, and relevant narrative reviews were considered for inclusion. Reference lists of selected articles were also screened to identify additional relevant publications. Studies were selected based on their relevance to the scope of the review, namely oral manifestations of chronic kidney disease, oral health in dialysis patients, oral complications of kidney transplantation, and dental management considerations in these patient populations.

Articles focusing primarily on non-oral manifestations of chronic kidney disease or lacking direct relevance to the review topic were excluded. Categories of publications and reason for exclusion are presented in Supplementary Table S2. As this was a narrative review, no formal systematic review methodology, risk-of-bias assessment, or quantitative synthesis was performed. The review was performed following the principles outlined by Sukhera [8].

## 3. Results

### 3.1. Xerostomia

Xerostomia is one of the most frequently reported oral manifestations in patients with CKD, including those undergoing dialysis. Observational studies and systematic reviews have reported prevalence estimates ranging from 41% to 56% in predialysis and hemodialysis populations. In addition, CKD and dialysis patients have been shown to exhibit significantly lower unstimulated and stimulated salivary flow rates than healthy controls (mean difference for unstimulated whole saliva: −0.11 mL/min; 95% CI −0.20 to −0.02) [9,10,11]. Objective hyposalivation is frequently accompanied by subjective complaints of dry mouth, which substantially impairs quality of life, affecting chewing, swallowing, taste, and speech [12]. Several factors have been associated with xerostomia in CKD, including advanced age, diabetes mellitus, hypertension, polypharmacy, and fluid intake restriction. Proposed mechanisms include salivary gland dysfunction, altered fluid balance, medication effects, and metabolic changes associated with declining renal function [11,12,13]. Studies have also shown that patients with end-stage renal disease often exhibit increased salivary pH and buffering capacity compared with healthy individuals. This finding has been attributed to elevated salivary urea concentrations resulting from impaired renal excretion; subsequent breakdown of urea into ammonia may contribute to a more alkaline oral environment [12]. Hemodialysis has been reported to acutely increase salivary flow and reduce salivary pH, although its long-term effect on xerostomia remains uncertain [11]. Several interventions have been investigated for the management of xerostomia in CKD and dialysis patients. Small clinical studies have reported improvements in salivary flow and patient-reported symptoms following the use of topical agents such as licorice mouthwash and thyme honey oral rinse [14,15]. Similarly, sodium and ultrafiltration profiling during haemodialysis has been associated with reductions in xerostomia symptoms and improvements in patient-reported quality of life [16]. However, the available evidence is limited by small sample sizes, short follow-up periods, and heterogeneity in outcome assessment. Mechanical stimulation techniques, including chewing gum and acupressure, have demonstrated modest short-term benefits, although evidence supporting sustained long-term efficacy remains limited [11].

Overall, current evidence supports xerostomia as a common and clinically relevant complication of CKD and dialysis. While several therapeutic approaches show promise, further well-designed studies are needed to establish their long-term effectiveness and determine optimal management strategies for this patient population.

### 3.2. Periodontal Disease

Periodontal disease and CKD are consistently associated in observational studies, and growing evidence suggests a potentially bidirectional relationship mediated by shared inflammatory, immunological, and metabolic pathways. CKD patients exhibit a higher prevalence and severity of periodontitis compared to healthy individuals, with meta-analyses demonstrating increased odds ratios for periodontitis in CKD populations and more advanced periodontal parameters such as clinical attachment loss and probing depth [17,18]. CKD-related factors—including uremic milieu, mineral bone disorders, metabolic acidosis, and impaired salivary flow—contribute to oral microbiota dysbiosis and increased susceptibility to periodontal tissue destruction. Conversely, periodontitis has been proposed as a chronic source of systemic inflammation that may contribute to the overall inflammatory burden observed in patients with CKD [19,20]. Observational and mechanistic studies have suggested that periodontal inflammation may be associated with declining renal function; however, a causal relationship has not been definitively established [17,21]. Non-surgical periodontal therapy has been shown to improve periodontal parameters and reduce systemic inflammatory markers in patients with CKD and periodontitis. However, evidence regarding its effect on renal function and CKD progression remains limited and inconclusive [18,22,23,24].

### 3.3. Gingival Overgrowth

**Figure 1:**
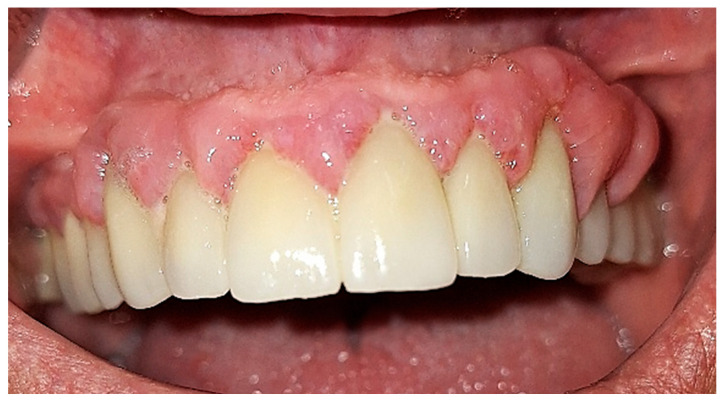
Gingival overgrowth after cyclosporine A therapy.

Gingival overgrowth is a well-recognized oral complication in patients with CKD, particularly among kidney transplant recipients receiving immunosuppressive therapy. The condition has been most frequently associated with cyclosporine A (CsA), although the underlying pathophysiological mechanisms have not been fully elucidated. Experimental studies suggest that CsA may promote gingival fibroblast proliferation and extracellular matrix accumulation through effects on cell-cycle regulation and pathways involved in epithelial-to-mesenchymal transition [25,26,27]. Clinical studies have reported a higher prevalence of gingival overgrowth among CsA-treated renal transplant recipients, with estimates ranging from 34% to 60%; however, prevalence rates vary considerably between studies and may be influenced by differences in patient populations, drug regimens, and diagnostic criteria [28,29,30,31]. Concomitant use of calcium channel blockers (especially nifedipine) further increases both the prevalence and severity of gingival overgrowth, with synergistic effects observed when combined with CsA (Figure 1). In contrast, tacrolimus-based regimens are associated with a significantly lower risk of gingival enlargement. Poor oral hygiene and pre-existing gingival inflammation have consistently been identified as risk factors for gingival overgrowth, with several studies demonstrating correlations between indices of gingival inflammation and lesion severity [28,29,30,31]. Gingival overgrowth can affect oral function, aesthetics, and access for dental care, sometimes necessitating surgical intervention, particularly in pediatric CKD populations [32]. Multidisciplinary management—including optimization of immunosuppressive regimens, avoidance of high-risk drug combinations, and rigorous oral hygiene is essential to mitigate risk and improve outcomes in CKD patients. However, the evidence supporting specific management protocols remains limited mainly to clinical experience, professional guidelines and smaller interventional studies [33].

### 3.4. Oral Infections

**Figure 2:**
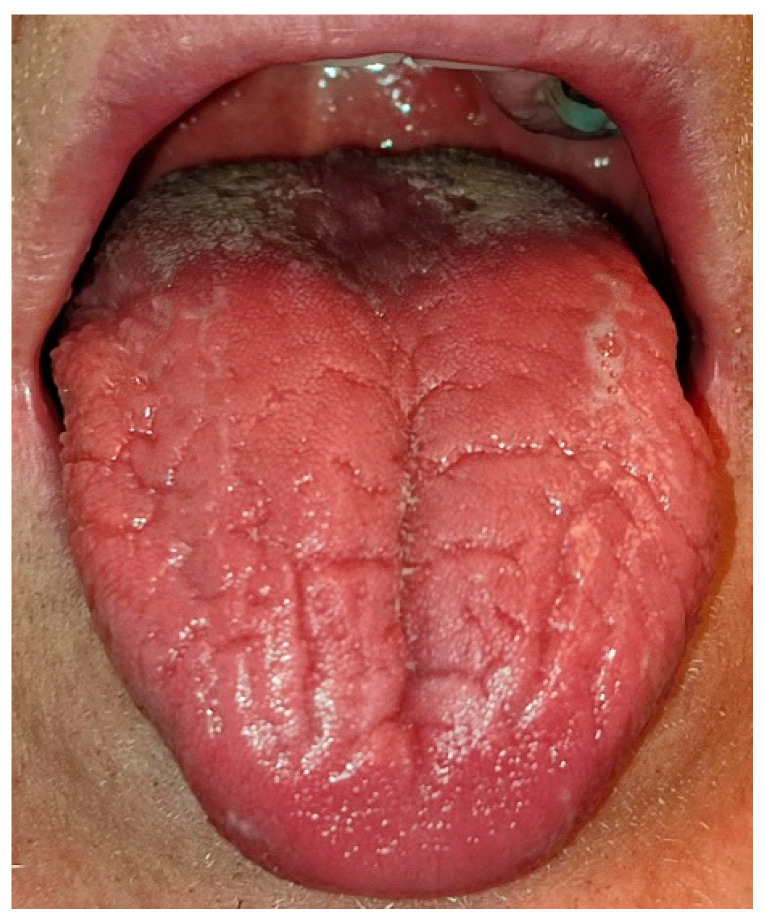
Oral candidiasis in a patient with end-stage kidney disease receiving haemodialysis.

Oral infections appear to be more common in patients with CKD, particularly in those with advanced disease and individuals undergoing dialysis. Several biological mechanisms have been proposed to explain this increased susceptibility, including immune dysfunction, reduced salivary flow, alterations in salivary composition, and changes in the oral microbiome associated with CKD (Figure 2) [4]. However, the relative contribution of these factors remains incompletely understood. The prevalence of apical periodontitis and periodontal disease is markedly higher in CKD patients on dialysis compared to those not on dialysis, with odds ratios for apical periodontitis and periodontal disease exceeding 2 and 6, respectively. Oral candidiasis is also more common in CKD, especially in the presence of diabetes or hypertension [34,35]. Emerging evidence suggests that the oral cavity of some patients with CKD may harbor multidrug-resistant microorganisms, potentially increasing the complexity of infection management [36]. In addition, observational studies have identified associations between oral infections, systemic inflammatory burden, protein-energy wasting, and adverse cardiovascular outcomes [34,37]. Although these findings support a possible link between oral health and systemic disease in CKD, the available evidence is predominantly observational and does not establish causality. Current evidence supports the importance of regular oral assessment and early management of oral infections in patients with CKD. While these interventions are widely recommended to improve oral health and quality of life, further prospective and interventional studies are needed to determine whether prevention and treatment of oral infections can influence renal outcomes or reduce systemic complications in this population [6].

### 3.5. Anemia and Mucosal Pallor

**Figure 3:**
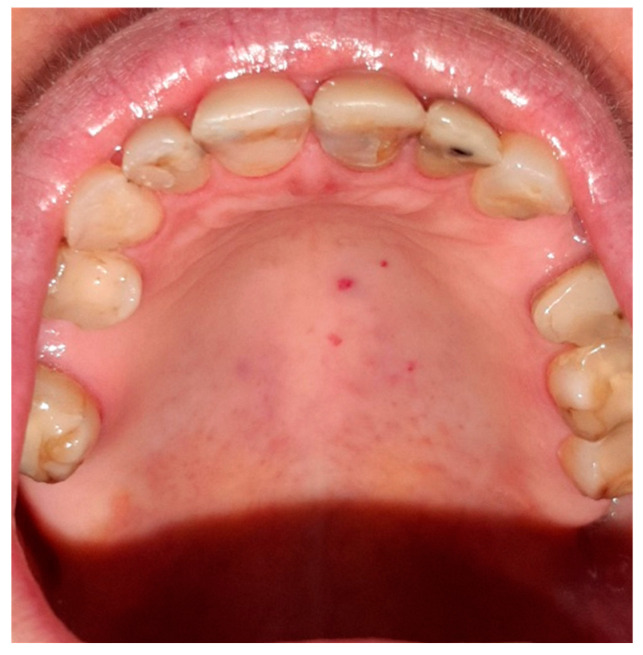
Oral mucosal pallor with advanced CKD-associated anemia.

Anemia is a frequent and clinically significant complication of CKD, with prevalence increasing as glomerular filtration rate declines and exceeding 50% in advanced stages. The pathogenesis is multifactorial and involves relative erythropoietin deficiency, iron dysregulation, chronic inflammation, and shortened red blood cell survival. Hepcidin-mediated iron sequestration and impaired iron absorption further contribute to functional iron deficiency, while absolute iron deficiency may result from inadequate intake, impaired absorption, or chronic blood loss [38,39,40,41,42,43]. Oral mucosal pallor is a frequently reported oral manifestation of anemia in patients with CKD and is thought to reflect reduced hemoglobin concentration and diminished tissue oxygenation (Figure 3). However, its diagnostic utility has not been specifically established in CKD populations and should be interpreted in conjunction with laboratory findings. Clinically, pallor is most observed on the labial and buccal mucosa and may be accompanied by mucosal atrophy in patients with severe or longstanding anemia. The presence of oral mucosal pallor may prompt further evaluation for anemia and its potential contributing factors. Management of anemia in CKD should follow established nephrology guidelines and may include iron supplementation and erythropoiesis-stimulating agents when clinically indicated. Although correction of anemia has been associated with improvements in symptoms and quality of life, the specific contribution of oral mucosal pallor to the early detection and management of anemia in CKD remains insufficiently studied [38,39,40,41,42,43].

### 3.6. Enamel Hypoplasia

Enamel hypoplasia and other developmental defects of enamel (DDEs) are frequently reported in children and adolescents with CKD. Observational studies have consistently demonstrated a higher prevalence of these defects in pediatric CKD populations than in healthy controls, with reported prevalence ranging from 19% to 88.8% in patients with CKD and from 3% to 44.2% in healthy individuals [44,45]. The pathogenesis of enamel hypoplasia in CKD is considered multifactorial. Experimental and clinical evidence suggests that disturbances in calcium and phosphate metabolism, vitamin D deficiency, secondary hyperparathyroidism, metabolic acidosis, malnutrition, and renal osteodystrophy may interfere with ameloblast function during tooth development. However, the relative contribution of individual factors remains incompletely understood [45,46,47].

Enamel hypoplasia in CKD is mostly seen in children with early-onset or advanced disease and can affect both primary and permanent dentition. The risk is particularly high when CKD onset occurs during the period of active enamel formation, with postnatal hypocalcemia identified as a potential risk factor. These enamel defects are associated with increased plaque and calculus accumulation, but paradoxically, children with CKD often have lower caries rates due to the alkaline oral environment resulting from uremia. However, available data are inconsistent, and the relationship between CKD, enamel defects, and caries risk remains incompletely understood. Following kidney transplantation, normalization of the oral environment may contribute to changes in caries risk, although evidence regarding this association remains limited [48,49]. Enamel hypoplasia and other DDEs negatively impact oral health-related quality of life and may complicate dental management in CKD patients. Regular dental surveillance and early preventive interventions are recommended for all children with CKD [50,51].

### 3.7. Oral Considerations in Kidney Transplant Recipients

#### 3.7.1. Pre-Transplant Dental Evaluation

A thorough dental evaluation is a significant part of preparing patients for kidney transplantation. Its main goal is to detect and address any potential sources of oral infection, which could pose serious systemic risks once immunosuppressive therapy begins. Untreated dental disease represents a potential risk for infection in transplant patients, with post-transplantation sepsis from suspected dental sources reported in 27% of surveyed centers [52]. Pre-transplant dental evaluation includes a detailed clinical examination, dental radiographs, and completion of all necessary treatments—such as managing active carious lesions, treating periodontal disease, and extracting teeth that cannot be restored [53,54]. Special attention should also be given to patients with poorly fitting dentures, which should be relined or adjusted to prevent trauma to the oral mucosa. Effective communication between the dental team and the nephrology or transplant team is required to ensure coordinated care, to review the patient’s overall health, and to determine the best timing for dental interventions. Many transplant centers recommend completing invasive dental procedures, including tooth extractions, before transplantation to allow adequate healing and reduce the potential risk of post-transplant infectious complications; however, this practice is supported primarily by expert consensus and clinical experience rather than comparative clinical trials [52]. Prior to performing such procedures, blood tests (including INR, PT, APTT, complete blood count and thrombocyte function tests) are advised to evaluate the risk of bleeding. Local hemostatic measures including sutures and hemostatic agents should be used when needed. Invasive dental procedures are commonly scheduled on non-dialysis days because of concerns regarding residual anticoagulant effects and bleeding risk associated with heparin administration during hemodialysis, although high-quality evidence supporting this practice remains limited [52,53,54].

#### 3.7.2. Post-Transplant Dental Management

The management of dental procedures in kidney transplant recipients requires careful timing and coordination with the transplant team, with distinct recommendations for the early post-transplant period when immunosuppression is most intense versus the later maintenance phase. During the first three months following kidney transplantation, when immunosuppressive therapy is typically most intensive and the risk of infectious complications is highest, elective dental treatment is generally deferred, and care is usually limited to urgent or emergency procedures. However, this recommendation is based primarily on expert consensus, transplant-center protocols, and clinical experience rather than direct evidence from randomized clinical trials [52,53,54]. Apart from the strongest immunosuppression, oral lesions are most common in the short term after transplantation. Sarmento et al. reported their prevalence increasing from 3.7% pre-transplant to 23.7% at 15–20 days and 25.7% at 45–60 days post-transplant, with ulcers and candidiasis being the most frequent complications [55]. Elective dental treatment is generally considered safer after stabilization of immunosuppressive therapy, typically three to six months after transplantation, although the optimal timing should be individualized in consultation with the transplant team. Even though no solid evidence (i.e., randomized controlled trials) exists, prophylactic antibiotics are usually recommended before invasive procedures. This practice is based primarily on expert consensus and survey data rather than high-quality randomized controlled trial evidence. A survey of US transplant centers found that 83% recommend prophylactic antibiotics prior to dental care, with 77% indicating it should be used for all dental procedures, whether invasive or not [52]. However, a prospective observational study of tooth extractions in stable kidney transplant recipients (more than 6 months post-transplant) found with no prophylactic antibiotic no statistical difference in postoperative complications or delayed healing compared to control patients, suggesting that stable transplant recipients may not have an increased risk of post-extraction complications [56].

#### 3.7.3. Long-Term Monitoring

Regular dental examinations every three to six months are commonly recommended for kidney transplant recipients to facilitate early detection and management of oral complications. However, this recommendation is based mainly on expert opinion and clinical guidelines, as the optimal surveillance interval has not been established through prospective studies. Removal of supra- and subgingival deposits, reinforcement of oral hygiene, and early intervention for any oral or dental lesions are essential components of long-term care.

### 3.8. Oral Cancer

**Figure 4:**
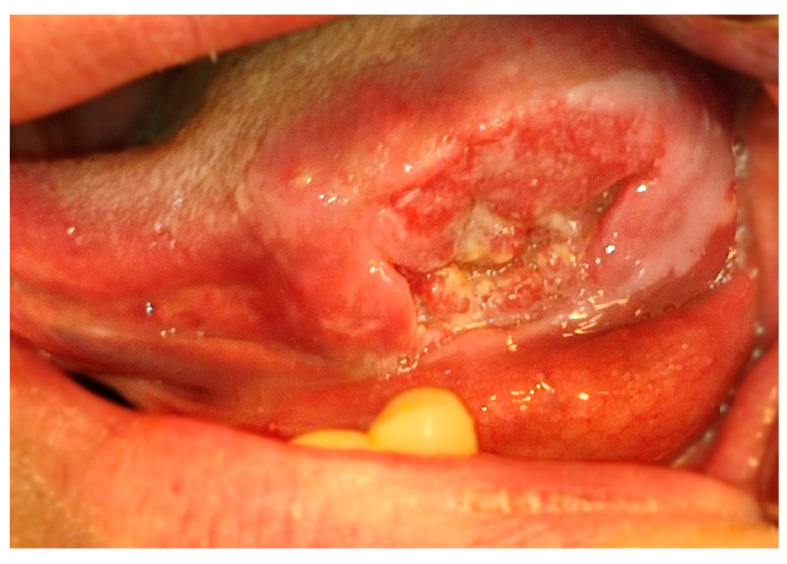
Oral cancer in a kidney transplant recipient receiving long-term immunosuppressive therapy.

Kidney transplant recipients have an increased risk of developing oral and lip cancers compared with the general population. Large registry-based studies have reported standardized incidence ratios of approximately 5–10 for oral cavity cancers and substantially higher risks for lip cancer, often exceeding 20-fold compared with the general population [57,58]. The risk is generally attributed to long-term immunosuppressive therapy, which may impair immune surveillance and reduce the ability to detect and eliminate dysplastic or malignant cells. However, the development of oral cancer in this population is likely multifactorial and may also be influenced by age, cumulative exposure to immunosuppressive agents, viral infections, ultraviolet radiation exposure (particularly for lip cancer), and other patient-related factors [59]. Cancer most commonly presents as a non-healing ulcer, a red and/or white patch with a speckled surface [60] (Figure 4). Early detection is critical because oral cancers in this population often present at a more advanced stage and have higher metastatic potential. Thorough lifelong oral examinations and patient education are crucial, as oral cancer can occur more than 10 or even 20 years after transplantation [61,62,63]. Any suspicious lesion must be promptly investigated and managed in close collaboration with the nephrology team.

### 3.9. Clinical Perspective: Oral Manifestations Across the CKD Continuum

The spectrum of oral manifestations observed in patients with CKD varies according to disease stage, treatment modality, patient age, and transplantation status. Consequently, oral findings should not be viewed as isolated complications but rather as part of a dynamic continuum reflecting the underlying pathophysiology of CKD and its treatment.

In the earlier stages of CKD, oral manifestations are primarily related to metabolic disturbances, chronic inflammation, and comorbid conditions such as diabetes and hypertension. Xerostomia, periodontal disease, oral infections, and anemia-related mucosal pallor are among the most frequently reported findings. As renal function declines, the cumulative effects of uremia, immune dysregulation, and altered salivary composition may further increase susceptibility to oral disease. Patients undergoing dialysis often experience additional oral complications. Fluid restriction, medication use, and dialysis-related physiological changes may exacerbate xerostomia and hyposalivation, negatively affecting oral function and quality of life. Furthermore, persistent systemic inflammation and immune dysfunction may contribute to a higher prevalence of periodontal disease and oral infections in hemodialysis patients [4,34,37].

The oral manifestations observed in pediatric patients with CKD differ from those seen in adults. Because kidney disease may interfere with normal tooth development, children are particularly susceptible to enamel hypoplasia and other developmental defects of enamel. These abnormalities may persist even after successful renal transplantation and can have long-term consequences for oral health and quality of life [44,45,46,47,48,49].

Following kidney transplantation, the pattern of oral disease shifts from manifestations primarily associated with renal dysfunction toward complications related to long-term immunosuppressive therapy. Gingival overgrowth, opportunistic infections, oral ulcerations, and an increased risk of oral malignancies become more prominent clinical concerns [5,57,58].

Taken together, these observations highlight the importance of adapting oral healthcare strategies to the patient’s position along the CKD continuum. Recognition of stage-specific oral manifestations may facilitate earlier diagnosis, targeted preventive interventions, and improved multidisciplinary management of CKD patients. Summary of oral manifestations across the CKD continuum along with their prevention and treatment modalities is presented in Table 1.

## 4. Discussion

The available evidence consistently demonstrates that oral complications are common in patients with CKD and kidney transplant recipients. However, the strength of evidence supporting individual associations and clinical recommendations varies considerably. Among the oral conditions discussed in this review, the association between CKD and periodontal disease has received the greatest research attention. Nevertheless, most available evidence remains observational, and causality has not been definitively established. While non-surgical periodontal therapy may reduce systemic inflammatory burden, evidence demonstrating a direct effect on CKD progression, renal function decline, or long-term clinical outcomes remains limited. Evidence supporting other oral manifestations is largely descriptive. Xerostomia, oral infections, anemia-related mucosal changes, gingival overgrowth, and developmental enamel defects are biologically plausible consequences of CKD-related metabolic disturbances, immune dysfunction, medication use, or transplantation-related immunosuppression. However, the available literature is heterogeneous, limiting direct comparisons between studies. In addition, these oral manifestations may be influenced by important confounding factors, including diabetes mellitus, hypertension, age, smoking, oral hygiene practices, and medication use. The review also highlights important differences across the CKD continuum. These observations underscore the importance of individualized dental management strategies based on disease stage and treatment modality.

It should be noted that many recommendations regarding the dental management of patients with CKD and kidney transplantation—particularly those related to pre-transplant dental assessment, timing of invasive dental procedures, antibiotic prophylaxis, and post-transplant surveillance—are supported by varying levels of evidence. Although these practices are widely accepted in clinical care, most are based on expert consensus, observational studies, and institutional protocols rather than high-quality randomized controlled trials.

This review has several limitations that need to be mentioned. As a narrative review, no formal risk-of-bias assessment or quantitative evidence synthesis was performed. The review was restricted to English-language publications indexed in PubMed/MEDLINE, which may have resulted in the omission of relevant studies indexed in other databases. In addition, the available literature is characterized by substantial heterogeneity in study design, patient populations, CKD stage, and outcome measures, limiting direct comparisons between studies. Furthermore, much of the evidence linking oral health and CKD outcomes is observational, precluding firm conclusions regarding causality. Future prospective studies and interventional trials are needed to better define the impact of oral health interventions on clinical outcomes in patients with CKD and kidney transplantation.

## 5. Conclusions

Oral manifestations are common in patients with CKD and kidney transplantation and may adversely affect quality of life, oral function, and overall health. Regular oral assessment and timely management of oral complications should be considered an integral part of multidisciplinary CKD care. Although associations between oral health and systemic outcomes are increasingly recognized, much of the available evidence remains observational, and the impact of oral health interventions on renal outcomes has yet to be fully established. Further prospective and interventional studies are needed to strengthen the evidence base and guide clinical care of these patients.

## Figures and Tables

**Table 1:** Oral manifestations across the chronic kidney disease continuum.

**Pretransplant and Dialysis**			
**Disease**	**Clinical Appearance**	**Complications**	**Treatment/Prevention**
**Xerostomia**	Subjective dry mouth often accompanied by objective hyposalivation Difficulty chewing and swallowing Taste alteration Speech impairment Reduced salivary flow	Increased caries risk, candidiasis, halitosis Difficulty wearing dentures Impaired nutrition Reduced quality of life	Saliva-stimulating/supportive measures Licorice mouthwash Thyme honey oral rinse Optimization of dialysis fluid/sodium and ultrafiltration profiling Hydration advice within nephrology limits Regular oral hygiene and caries prevention
**Periodontal Disease**	Increased prevalence and severity of periodontitis Deeper periodontal pockets Clinical attachment loss Gingival inflammation Bleeding Bone loss Tooth mobility in advanced cases	Tooth loss Worsening systemic inflammation Possible acceleration of CKD progression Increased cardiovascular risk Impaired mastication	Non-surgical periodontal therapy (scaling and root planing) Meticulous plaque control Regular periodontal maintenance Smoking cessation Management of diabetes and systemic inflammation Close dental follow-up
**Anemia and Mucosal Pallor**	Pale oral mucosa, especially labial and buccal mucosa May coexist with mucosal atrophy Pallor correlates with severity of anemia	Fatigue Delayed healing Reduced exercise tolerance Cardiovascular burden Mucosal atrophy/ulceration in severe cases	Investigate anemia causes (iron deficiency, EPO deficiency, inflammation) Oral or IV iron supplementation Erythropoiesis-stimulating agents when indicated Medical management of CKD anemia
**Pediatric chronic kidney disease**			
**Disease**	**Clinical Appearance**	**Complications**	**Treatment/Prevention**
**Enamel Hypoplasia**	Developmental enamel defects in children/adolescents Grooves Discoloration Reduced enamel hardness Sensitivity Tooth wear Plaque retention	Tooth sensitivity Plaque retention Increased caries risk	Early preventive dental care Fluoride therapy Fissure sealants Desensitizing agents Restorative treatment when needed Regular surveillance during growth and after transplantation
**Post transplant**			
**Disease**	**Clinical Appearance**	**Complications**	**Treatment/Prevention**
**Gingival Overgrowth**	Enlarged fibrotic gingiva, especially papillary/interdental areas Pseudopockets Impaired aesthetics, speech, mastication and oral hygiene access Common in cyclosporine users, worsened with calcium channel blockers	Plaque retention Secondary periodontitis Impaired speech/mastication Aesthetic concern Difficult oral hygiene	Improve oral hygiene and inflammation control Review medications with physician (switch from cyclosporine if possible, avoid high-risk combinations) Professional cleanings Gingivectomy/periodontal surgery if severe
**Oral Infections**	Increased frequency/severity of candidiasis Periodontal infections Apical periodontitis Possible ulceration Erythema White plaques Pain Swelling Abscesses	Pain Systemic spread of infection Increased inflammation Hospitalization risk Nutritional decline Possible worsening CKD outcomes	Routine dental screening Early treatment of caries/endodontic lesions Periodontal therapy Antifungal treatment when indicated Strict oral hygiene Multidisciplinary management with nephrology team
**Lip and oral cavity cancer**	Speckled red-and-white patches (erythroleukoplakia) Non-healing ulceration Indurated or exophytic mucosal lesion Most commonly affects the lips and tongue Asymptomatic in early stages	Local tissue destruction Pain, dysphagia, and impaired oral function Cervical lymph node metastasis Delayed diagnosis due to subtle early presentation Reduced quality of life Increased morbidity and mortality in advanced disease	Regular oral examinations Patient education regarding self-monitoring and early warning signs Prompt biopsy and specialist evaluation of suspicious lesions Reduction of modifiable risk factors (tobacco, alcohol, excessive sun exposure for lip cancer)

## Data Availability

No new data were created or analyzed in this study.

## Supplementary Materials

The following supporting information can be downloaded at: https://www.mdpi.com/article/10.3390/jcm15134940/s1, Table S1: Literature search strategy; Table S2: Categories of publications excluded during literature selection and reasons for exclusion.

## Abbreviations

The following abbreviations are used in this manuscript:

CKD	Chronic kidney disease
ESRD	End-stage renal disease
CsA	Cyclosporine A
DDE	Development defect of enamel
