# Supplementary material for: The Impact of Chronic Kidney Disease on Oral Health: A Narrative Review
Source: J Clin Med. 2026 Jun 25;15(13):4940. doi: 10.3390/jcm15134940 (PMC13361670; doi:10.3390/jcm15134940)
Supplement: Supplementary file 1 [file jcm-15-04940-s001.zip › jcm-4344455-supplementary.pdf]

Supplementary Table S1. Literature search strategy

**Item**	**Description**
Database searched	PubMed/MEDLINE
Date of search	March 2026
Publication period	January 2000 – March 2026
Language restriction	English
Types of publications considered	Original research articles, systematic reviews, meta-analyses, narrative reviews, clinical guidelines, and consensus statements
Population of interest	Patients with chronic kidney disease, end-stage renal disease, dialysis patients, and kidney transplant recipients
Main topics of interest	Oral manifestations of CKD, oral diseases, oral infections, dental abnormalities, and dental management
Search terms	"chronic kidney disease", "CKD", "end-stage renal disease", "ESRD", "hemodialysis", "haemodialysis", "dialysis", "kidney transplant", "renal transplant", "oral lesions", "oral mucosa", "oral disease", "periodontitis", "gingivitis", "gingival hyperplasia", "gingival enlargement", "xerostomia", "dry mouth", "hyposalivation", "stomatitis", "candidiasis", "Candida", "oral infection", "mucosal pallor", "enamel hypoplasia", “developmental defects of enamel”, "dental caries", “lip cancer”, “oral cancer” and “oral malignancies”
Search approach	Keywords were searched in relevant combinations using Boolean operators (AND, OR).
Inclusion criteria	Publications addressing oral manifestations, oral health status, oral complications, or dental management of patients with CKD, dialysis, or kidney transplantation.
Exclusion criteria	Publications unrelated to oral health, studies focused exclusively on acute kidney injury, non-human studies without direct clinical relevance, non-English publications, and reports lacking relevance to the review objectives.
Additional search methods	Reference lists of relevant articles were manually screened to identify additional eligible publications.
Evidence synthesis	Narrative synthesis of findings organized according to major oral manifestations and clinical management considerations.

Supplementary Table S2. Categories of publications excluded during literature selection and reasons for exclusion

**Category of publication**	**Reason for exclusion**
Studies focusing exclusively on non-oral manifestations of chronic kidney disease	Outside the scope of the review, which focused on oral health and dental management.
Studies investigating acute kidney injury rather than chronic kidney disease	Did not address the target patient population.
Animal studies	The review focused on clinical findings and management in human patients.

Category of publication	Reason for exclusion
In vitro studies without direct clinical relevance	Limited applicability to oral manifestations and dental care in CKD patients.
Publications focusing exclusively on nephrological outcomes without assessment of oral health	Outside the predefined review objectives.
Articles addressing oral health in systemic diseases other than CKD without separate CKD subgroup analysis	Unable to extract CKD-specific conclusions.
Conference abstracts, letters to the editor, editorials, and expert opinions without original data or substantial review content	Insufficient methodological detail or evidence synthesis.
Non-English publications	Excluded according to the predefined eligibility criteria.
Studies with insufficient relevance to oral manifestations, oral diseases, or dental management in CKD patients	Did not contribute directly to the aims of the review.
